# Supplementary material for: Provider and administrator-level perspectives on strategies to reduce fear and improve patient trust in the emergency department in times of heightened immigration enforcement
Source: PLoS One. 2021 Sep 10;16(9):e0256073. doi: 10.1371/journal.pone.0256073 (PMC8432754; doi:10.1371/journal.pone.0256073)
Supplement: S1 Appendix — (DOCX) [file pone.0256073.s001.docx]

1. How long have you been a clinician/administrator?
2. Existing system/structure
   1. How aware are providers of patients’ immigration status? How is this information communicated, if at all?
   2. What policies or protocols, if any, currently exist in your ED to help make all patients, including undocumented patients, feel safe?
   3. What do you do, or have seen others do, to help make all patients feel safe and build trust in the ED?
   4. What training, if any, have you received on patients’ rights in the Emergency Room (for example, patient rights if immigration enforcement were to visit the ED)?
3. Timeline of events
   1. Over the past 5 years, has your ED undergone any policy changes or other efforts to change practices in the ED to create a more inclusive environment for all, including undocumented patients?
      1. What inspired this policy/protocol change?
      2. How has [the policy change] impacted your patients, if at all?
4. Impact on patients
   1. How have presidential statements and campaign rhetoric about immigrants impacted your patients, if at all?
   2. Have you experienced patients delaying the time they took to access emergency care due to fear of discovery for themselves or loved ones?
      1. If yes: Did the delay, or could have the delay, impact(ed) their outcome or prognosis?
5. Future system/structure changes
   1. If you had a magic wand and could make any changes in your ED to address undocumented patients’ fear of discovery and build safety and trust, what would it be?
