# Supplementary material for: Provider and administrator-level perspectives on strategies to reduce fear and improve patient trust in the emergency department in times of heightened immigration enforcement
Source: PLoS One. 2021 Sep 10;16(9):e0256073. doi: 10.1371/journal.pone.0256073 (PMC8432754; doi:10.1371/journal.pone.0256073)
Supplement: S2 Appendix — (DOCX) [file pone.0256073.s002.docx]

1. How long have you been a clinician/administrator?
2. Existing system/structure
   1. What policies or protocols, if any, currently exist in your ED to help make all patients, including undocumented patients, feel safe?
   2. How is information about immigration status communicated, if at all?
   3. What training, if any, has your department received on patients’ rights in the Emergency Room (for example, patient rights if immigration enforcement were to visit the ED).
   4. What policies, if any, do you have in place if immigration enforcement were to visit your ED/hospital?
3. Timeline of events
   1. Over the past 5 years, has your ED undergone any policy changes or other efforts to change practices in the ED to create a more inclusive environment for all, including undocumented patients?
      1. What inspired this policy/protocol change?
      2. How has [the policy change] impacted your hospital, if at all?
4. Future system/structure changes
   1. What if any, future system-level changes, are you planning on implementing within the next year to alleviate undocumented patient fear and build trust in the ED?
   2. If you had a magic wand and could make any changes in your ED to address undocumented patients’ fear of discovery and build safety and trust, what would it be?

*If there is time and the administrator has clinical experience they would like to share (ask the following questions derived from the provider script).*

1. Impact on patients
   1. How have presidential statements and campaign rhetoric about immigrants impacted your patients, if at all?
   2. Have you experienced patients delaying the time they took to access emergency care due to fear of discovery for themselves or loved ones?
      1. If yes: Did the delay, or could have the delay, impact(ed) their outcome or prognosis?
