# Supplementary material for: Provider and administrator-level perspectives on strategies to reduce fear and improve patient trust in the emergency department in times of heightened immigration enforcement
Source: PLoS One. 2021 Sep 10;16(9):e0256073. doi: 10.1371/journal.pone.0256073 (PMC8432754; doi:10.1371/journal.pone.0256073)
Supplement: S1 Table — (DOCX) [file pone.0256073.s003.docx]

| **Theme** | **Exemplar quotations** |
| --- | --- |
| Theme 1: A majority of participants identify at least one of two hospital policies that help undocumented patients feel safe. | “The communication from leadership says that all staff do not have to cooperate and are under no obligation to release any information.” Administrator  “Period. Like we can’t deny that we are funded and governed by the federal and state governments, we are. But...we’re part of the State of California, and therefore, we can say no immigration agents are allowed on our land, and if you’re found here, the sheriffs are gonna arrest you.” Nurse  “We are told that the patients have a right to – that this is a safe place for patients to get services, and [Immigration and Customs Enforcement] ICE is not to come and disrupt that. That has been communicated to us.” Social worker  “We have several policies around as providers, how we communicate with Customs and Border Protection. If they were to come on campus, we are not authorized to communicate with them about a patient's status. And we also have signage around our emergency department, that just talks about – And around the hospital, for that matter, that talks about our sanctuary status...patient should feel comfortable seeking care from our hospital system without fear of their immigration status being shared with anyone outside of the medical staff.” Physician |
| Theme 2: Recent policy changes specific to undocumented patients include increased inclusive messaging and further restriction on immigration enforcement around and inside of the hospital. Other non-policy changes include increased staff awareness about issues impacting undocumented patients and changes in patient resources and work flows. | “I believe there’s a lot of signs now that are welcoming the undocumented individuals, also trying to remove the fear factor that they can come here and seek medical help without any repercussion. So, it’s visible, but then there’s still that paranoia and fear. I mean you are talking about their safety and their livelihood, the possibility of being sent back to their country, so they are not gonna totally let their guards down, and I understand that.” Social worker  “Well, I know that [county] has a policy that [Immigration and Naturalization Services] INS is not allowed, or Homeland Security now is not allowed into patient care areas without a warrant. And so, that is supposedly supposed to help although that doesn’t prevent them from parking outside of the hospital. But that should help in the fact that patients don’t have to worry about being detained while at one of the hospitals, but I don’t think that’s very well publicized to patients. So, I’m not sure if they really know that.” Physician  “I developed a lot of programs for the emergency room because we didn’t even have a social worker assigned to the emergency room until I got there. So, now we have 24/7 coverage. We’ve developed a program for the homeless for substance use, for taking care of grief...provide support for family.” Administrator |
| Theme 3: There are limited previous and future policies specific to undocumented patients and administrators defend this. | “I struggle a little bit with these questions...we are a community hospital of diversity and of equity...clearly, I think everyone in their own interaction with the patients do bring up certain issues and topics that they might recognize. Let’s say sex trafficking, for instance. Those are things that come up as we are all in tuned to recognize comments, signs, or symptoms or red flags that may come up....whether that has to be in a policy or protocol, I’m not sure how good that would do. I’m not sure how when that’s disseminated or read by those providers. But, I certainly would welcome any sort of that.” Administrator  “We’re a county facility and we work with this population. I mean we’ve always worked with this population and we’ve always worked with the underserved; we’ve always worked with the undocumented and the homeless. So, I think it is sort of ingrained that I think you have to have sort of that kind of mindset to work at a county facility...so, I don’t think we’ve made any major changes. I think we’ve always tried to accommodate each individual with whatever services we could get for them.” Administrator  “Well I know that we have changed the way we talk about gender and just inclusivity in that way. And that has been a real obvious change in terms of how registration asks questions – but I don’t know specifically – to immigration status if that is something that’s changed.” Social worker  “I can’t think of any practice and policy changes...I feel like we’ve always treated everybody – that’s always been the underlying fabric of our organization… Perhaps there was some changes, but I just was not aware of actually formal change in policy.” Nurse |
| Theme 4: Current training on policies and patients’ rights is limited. In general, communication about policies is top-down, email-based, and disparate. | “Very minimal training, none...All we've had is...one or two emails that were sent out maybe a few months back when the Trump administration said that they were going to deport people, and the email had an attachment and that was the only training. And even at that time it was like a handout that we could give to patients.” Nurse  “I am not aware of any training...we may talk about it, but nothing specific to our institution or what our policy is on immigration enforcement in the [Emergency Room] ER.” Administrator  “Sometimes I feel like communication between upper management, administration, and with like directly with nursing staff in particular is really poor in terms of like … I feel like the doctors have their own kind of like world and the residents and the nurses are separate. And, I mean, I feel I see the signs you know. Oh, immigrants are welcome and like there’s news articles about it being like a sanctuary hospital. But, like I didn’t even know we had a human rights clinic...I just feel like there’s such lack of communication between like all the different things that are happening in [hospital].” Nurse  “No formal training. I happen to have an interest in medical ethics and so I've done a lot of reading and had a lot of meetings and stuff like that, related to that. So, I had a lot of discussions and kind of extracurricular stuff, but nothing formal.” Physician  “We’ve had, like, two trainings in the past, very minimal...one of the concerns was what if, like, an [Immigration and Customs Enforcement] ICE agent or, you know, was outside in the parking lot and they tried to arrest someone, or what – what should we be doing as – as staff members if they – they, you know, if they tried to stop people? And then as far as inside the hospital, just, you know, we can’t give them any type of information no matter what. They would just have to call the hospital administration.” Social worker |
| Theme 5: There is significant uncertainty across all provider-types on policies, laws, and the roles of staff. | “We are a sanctuary city; however, you want to define it. Are we really sanctuary? I don’t know.” Administrator  “I think there are [policies], but I have not read them recently, so I don’t know. I’d have to go back and look.” Administrator  “Well, I’m not sure of any existing policy per se. I mean, I know that any patient that presents to be emergency or – I mean, they can’t be asked their immigration status. They, you know – I don’t know if it’s written down anywhere, but we don’t typically ask them if they are here legally or illegally.” Nurse  “Because of public charge, there was an effort made by individuals in the department to emphasize the fact that public charge doesn’t really apply, or when it does apply to our patient population and what to say to patients and there’s some instructions and stuff that we can give. But I don’t know how that reaches the actual patient...and I’m not sure how many providers are aware of that...then again, I don’t read all my emails. So, there might have been something in place.” Physician  “No [training]. And, I’ve actually wondered about that specifically. Like, what am I allowed to like do legally and like are we allowed to not have to like bar [Immigration and Customs Enforcement] ICE from coming into the hospital?” Nurse  “Domestic violence, I think, is a little more clear, at least to me. A lot of patients aren’t aware of that, but we’re able to assure them more. But, I don’t want to assure someone that because they were assaulted on [public transit], that if we call the police, that there won’t be an impact on them, because I don’t know that to be true. So, maybe that’s the thing I should find out. But, I would not encourage that person who was fearful to call the police, because I don’t feel confident in what would happen; whereas a domestic violence patient, I would.” Social worker |
| Theme 6: Status is asked about and documented inconsistently across sites and providers. Although status doesn’t impact clinical care, it comes up in a handful of clinical scenarios. | “Yes, it is communicated because it does – it’s gonna depend on what kind of services we can provide...if somebody is having difficulty with the social determinants of health issue, then my workers pretty much just straight out ask if they are...we don’t ask them if they’re undocumented…we use the term a person without documentation. So, it doesn’t label them as that’s the whole person...if somebody needs a placement in a skilled nursing facility, we’re gonna need to have insurance. If somebody needs congregate or board and care or things like that, that kind of placement comes with getting money. It doesn’t come through the insurance. So, it’s important we know if they can get social security or disability or any of that. So, if you’re undocumented you don’t get any of it. So, it’s important for us to know right up in front what we’re dealing with.” Administrator  “I guess I would say the whole emergency room is my team. Information could be communicated in a note, patient's undocumented; it can be verbal, over the phone. And, so I think that is probably how the information gets to me.” Social worker  “I’m fearful that if I write in a chart that someone’s undocumented, that that could be discoverable in some other setting that could have ramifications for the patient.” Physician  “I think there’s assumptions made probably, but I don’t … I personally am not aware of somebody’s like legal status and I never ask.” Nurse |
| Theme 7: Providers have cared for undocumented patients who have experienced barriers to accessing emergency care and have delayed care due to fear of discovery. | “Yeah. I feel like I’ve seen several people who … well, several women who had been being in like really abusive situations for a long time and were afraid of the police. And then, like a couple of people who had bad infections that had been going on for a long time...And then, I worry too like with COVID-19 if people aren’t seeking out care because I feel like we’re starting to see it pop up more in like our community here including the Latino community.” Nurse  “There was another incident where a woman came in with tooth pain. And by the time she got there she was in severe pain. And crying and when I saw her and talked to her, I was like oh, just trying to understand why she hadn’t come in sooner...she was like, yeah. I came here two days ago, and I saw immigration, a vehicle outside of the hospital parked outside of the emergency department and it scared me so much that I went home and I just thought I could get through it because I didn’t wanna go in there. I was scared because I’m undocumented.” Physician  “So, there was a guy who actually is homeless. I think he had some substance abuse, and...he has a wound on his foot, probably has diabetes but has had that wound for almost a year and was scared to go get it treated because he was scared of being deported.” Social worker |
| Theme 8: Providers describe efforts to build safety and trust and considered their roles as protecting and supporting undocumented patients. Some even independently learn about immigration policies/ethics. | “I think consistent things that I'll do is either ask the patient or the family that's with them, ‘Is there anything I can do to help you be more comfortable while you're here?’ Then especially people who they seem like they're stressed out or scared or fearful...I often will tell people, ‘You're safe with me. You're safe here and you know it's my job to protect you’...No formal training. I happen to have an interest in medical ethics and so I've done a lot of reading and had a lot of meetings and stuff like that, related to that. So, I had a lot of discussions and kind of extracurricular stuff, but nothing formal.” Physician  “So, first of all, we tell them that their medical concern is valid. And we – in the emergency room, we do feel – they have to be secure and we do not report their status to anyone. It’s all confidential. For example, I don’t speak Spanish. So, if a Spanish person comes, what I do is actually I’ll get a [Certified Nursing Assistant] CNA who speaks Spanish or the RN or I use the translator services. And I tell them that everything is confidential, and nothing will be shared with anyone. I don’t definitely say with authorities or use words like that. But I will say something like whatever they tell me is confidential and the same thing the translator says, whatever I speak in English will be translated to them in Spanish and everything is confidential.” Nurse  “Usually what a lot of us would do is be very clear that we have no connection with [Immigration and Customs Enforcement] ICE. You have to be very clear. You have to say, you know, “Hey, we are not the police. We are only concerned about your medical needs and what else we can do to sustain you and your family.” So, you have to – because they are always gonna have that fear factor, but you can kind of help them relax and open up a little bit more because they are watching security walk around. They are looking at the deputies walk around, and so and then there are people out there who’ve threatened to call Immigrations on them. So that’s why I think they come here later at night.” Social worker |
| Theme 9: Interviewees suggest a wide range of future changes ranging from individual to structural changes. | “I would do more of a formal training...include just what we have already set in place...also come into the psychological factor of why it can be hard for them to seek help...how to try and alleviate any psychological anxiety or fear on their part...not just nursing staff, but clerks or janitors...to try to make the patient be more comfortable, feel this is a safe place for them.” Administrator    “We ask people about, for identification, whether that’s to like get them in the door right away or like for registration, it should be really clear that this is in no way are we, does their immigration status affect the healthcare they’re gonna receive and we are not going to be speaking to [Immigration and Customs Enforcement] ICE or immigration law enforcement, so. And, more signage in different languages…not just Spanish. I mean, immigrants from all over the world.” Nurse  “So, I think if you really want to make change, you need to reach out to the community, to the people who live in those communities, the churches or the places where people have trust already. Because I think having someone trust a hospital or a government-run hospital is not as easy as if the organization that you normally go to every Sunday that you trust. It's right there, you hear that these places are actually safe…even if the hospital has a big sign that says, "Immigrants are welcome", there's still a lot of distrust. There's still people that will say, "Well I went to the hospital and I heard so and so this." And there's always things that I think are anecdotal that might undermine hospital-wide.” Physician  “I think it would be education. Not only for our staff here, maybe on how to better interact with undocumented people, but also to ease their fear, and sometimes how to word things. Sometimes, we’re telling patients the correct information, but we’re wording it in not the best manner, so it scares people. And also, education for the patients to be really educated on what the hospital’s role is, and what our role is when it comes to immigration services and also for the healthcare piece... maybe more community resources because undocumented people, unfortunately, do not qualify for the majority of things. For example, even for basic care, just primary care, it’s so difficult for them to access those resources. If we can get better primary care clinics set up for undocumented people where they could get full care, I think it would change things.” Social worker |
