# Supplementary material for: Provider and administrator-level perspectives on strategies to reduce fear and improve patient trust in the emergency department in times of heightened immigration enforcement
Source: PLoS One. 2021 Sep 10;16(9):e0256073. doi: 10.1371/journal.pone.0256073 (PMC8432754; doi:10.1371/journal.pone.0256073)
Supplement: Attachment — Submitted filename: Response to ReviewersPONE-D-20-28725.doc [file pone.0256073.s004.doc]

**Reviewer 1:**

Thank you for the opportunity to review this manuscript. It is excellent. The authors provide a thorough overview of the challenges of ensuring the safety of undocumented immigrants in the ED, and their methods are well-described and appropriate. The only minor revisions that I believe this manuscript could use would be:

(1) a definition/explanation of the term "public charge," which may not be familiar to all readers

*Thank you for this recommendation. We have added clarification to what public charge is.*

(2) including/incorporating 1-2 illustrative quotations per theme in the findings section. Although the themes are clearly described and this section is strong without the quotations, adding them to the text (rather than/in addition to having one per theme in Table 1) would add richness to the manuscript and presentation of findings.

*Thank you for this comment. We have integrated quotes into the text of the results and have removed Table 1. Additional quotations are still available in the supplemental table 1.*

(3) perhaps adding a reference to the toolkit developed from Saadi's study described on page 16, which includes many of the suggestions the authors describe (doctorsforimmigrants.com)

This paper presents an important contribution to the literature on the safety of undocumented immigrants in health care settings.

*Thank you for this recommendation. We have added a note, both in the text and references, about Dr. Saadi’s toolkit.*

**Reviewer 2:**

This is a worthwhile study and I appreciate your doing it. I do have a few questions and comments.

First, it appears that some of the authors are emergency physicians, who presumably have experience caring for undocumented people and likely came to this with some preconceptions and opinions. This should be acknowledged and discussed in the methods.

*Thank you for this comment. We have noted this in our limitations section and provided clarification that one of the two researchers who conducted the analysis is tied to emergency medicine while the other is not.*

In the introduction, you say “These disparities were exacerbated under the Affordable Care Act due to the exclusion of undocumented immigrants from marketplace health care plans and Medicaid expansion.” This observation seems misleading as phrased because undocumented people were not harmed by the ACA, they just didn’t benefit from it. In fact some have family members, notably their children, who did benefit.

*Thank you for this comment. We have altered the statement to now read as more neutral.*

On page 6, you say you used a combination of in-person recruitment and snowball sampling. I don’t understand why you used snowball sampling. You evidently had the cooperation of the ED directors and presumably access to complete staff rosters. Why not use random or purposive sampling? It seems possible that respondents who referred others to you would have chosen like-minded people, which could affect the representativeness of your respondents.

*Thank you for this comment. We added clarification about the snowball sampling. Recruitment was mainly in person/email recruitment and snowball sampling was utilized when there was limited responsiveness. Random sampling is not necessarily a goal of qualitative research, given that the goal is not to produce research that generalizes to all ED physicians, but to identify common and outlying patterns and themes. We focus on examining in-depth provider/admin perspectives with smaller sample sizes (compared to quantitative research). We have added acknowledgment of the limitations of snowball sampling in the limitations section.*

On page 10 you write “informing patients about changes in public charge . . . “ I think I know what you mean but you should explain this.

*Thank you for this comment. We have added clarification to what public charge is.*

Regarding the reference to ankle bracelets on page 12, I am guessing this refers to asylum seekers who have been released pending a hearing? You should explain. If so, ICE is already aware of these people and their status so there would not seem to be any particular danger to them from seeking care, although the public charge issue might be relevant, if I understand this correctly.

*Thank you for this comment. We have added further clarification in the text about the ankle bracelets. This was in response to how providers learned about patients’ status (and not about patients seeking care).*

Regarding documentation of status, I note that I once worked for a community-based behavioral health provider that targeted a particular immigrant community. If we had to document status for some reason, we did it in a way that could not be detected even if the records were subject to a court order. If you can, you might make it more clear how status was documented in those cases where providers deemed it necessary.

*Thank you for this insight. Unfortunately we don’t have further details around how exactly it was documented, so have added this clarification to the manuscript.*

On page 10 you write “Only one physician mentioned interacting with immigration enforcement, for the sole purpose of providing medical care.” Please explain. Why was it necessary to interact with immigration enforcement in order to provide medical care?

*Thank you for this comment. We have added more context around this scenario (patient was under custody of immigration enforcement).*

Regarding the limitations, why are nurses and social workers difficult to reach? In my experience it is must more difficult to get physicians to take the time to talk with me. I have no trouble interviewing nurses or social workers.

*Thank you for this comment. We have removed this statement and replaced it with “busy frontline providers” which more accurately expresses our recruitment experience. The emergency doctors often had more flexibility in their schedules and were able to accommodate interviews.*

I like the tables, but make sure to format so they fit on one page.

*Thank you for this comment. We have formatted it to fit on one page.*

**Reviewer 3:**

Thank you for the opportunity to review this manuscript describing a qualitative research project conducted in three California emergency departments. The focus of the project is to explore the experience of undocumented patients and how staff can interact with them to provide care while mitigating fear and anxiety. The study is conducted among 41 participants including a range of emergency Department providers as well as administration. The researchers take valuable measures to ensure that the information is gathered without any hint of threat or coercion and adhere to sound principles of qualitative research.The main findings of this work highlight 9 themes that cover a range of topics including existing policies that address undocumented patients as well as gaps in educational programs that optimize care for this vulnerable and marginalized population. The project goes on to draw from these conclusions and generate useful guidance for next steps that can address the issues that were identified. Overall, I think this is a very well written and informative manuscript. While based on the California experience its findings have applicability in numerous jurisdictions. I also commend the authors for working in an area that highlights in equities and doing so in wave one of the covid pandemic is a remarkable accomplishment.

*Thank you for this comment.*

As a reviewer coming from a Canadian perspective I do have a number of suggestions that could make the manuscript more relevant to the non US reader. For example are the paper does not provide clear information related to how undocumented patience might be treated any differently in an emergency department than those who are legal. More information as to the range of complaints and pathologies that will bring an undocumented patient to the emergency department and which are unique from the general population would be appreciated. Similarly additional insight into the experience of the undocumented emergency department patient would provide useful additional context. Finally a more elaboration on US specific policies for example hippa and emtala might make the manuscript more accessible to the global reader.

*Thank you for this recommendation. We have added a brief acknowledgement of the international landscape in the limitations. You highlight excellent questions. There is unfortunately scarce information in the literature in regards to pathologies among those who are undocumented vs those that are not, and differences in treatment/experience. These questions are beyond the scope of our study, and we have focused on describing the well established phenomenon of changes in healthcare access in the setting of anti-immigrant rhetoric. There is a description of EMTALA in paragraph 3 of the introduction and we have included elaboration about HIPAA.*

**Reviewer 4:**

This is an interesting work for health policy makers.

I have some concerns:

1- How did COVID-19 pandemic situation affected the study and the responses of ED individuals to your interview questions? I have a concern that these results might be biased by the COVID-19 situation.

*Thank you for this comment. This is a valid concern. We have added clarification around the very little impact COVID-19 had on our study (other than conducting it via phone). The interviews before and after the start of COVID did not have differences in themes.*

2- How did you select the recruited individuals? Because without random sampling, this sample will not be representative.

*Thank you for this comment. We added clarification about the snowball sampling. Recruitment was mainly in person/email recruitment and snowball sampling was utilized when there was limited responsiveness. Random sampling is not necessarily a goal of qualitative research, given that the goal is not to produce research that generalizes to all ED physicians, but to identify common and outlying patterns and themes. We focus on examining in-depth provider/admin perspectives with smaller sample sizes (compared to quantitative research). We have added acknowledgment of the limitations of snowball sampling in the limitations section.*

3- How your findings will differ from other states and other countries?

*Thank you for this comment. We have included a brief discussion in the limitations.*

Overall, the work is good and publishable.

*Thank you for this comment.*

**Reviewer 5:**

This is a nicely written, qualitative piece which examines ED strategies to reduce fear and improve patient trust in times of heightened immigration enforcements. The study is unique and is important to form ED practice.

Significance: Is large given vulnerability of immigrant populations, particularly in the times of the pandemic,

Methods: While there were 40 subjects, there was a nice mix of different types of practitioners and the authors obtained saturation of themes.

Generalizablity: This is a qualitative survey of administrators, nurses and physicians in 3 safety net hospitals in California that care for a large number of immigrants. Although California has unique laws for immigrants, it also has the largest proportion of immigrants. This study can inform other California sites about strategies to help immigrants most readily but also can provide information to other EDs interested in this advocacy.

*Thank you for this comment.*

**Journal requirements:**

1. Please ensure that your manuscript meets PLOS ONE's style requirements, including those for file naming. The PLOS ONE style templates can be found at

[https://journals.plos.org/plosone/s/file?id=wjVg/PLOSOne_formatting_sample_main_body.pdf](https://urldefense.com/v3/__https://journals.plos.org/plosone/s/file?id=wjVg*PLOSOne_formatting_sample_main_body.pdf__;Lw!!LQC6Cpwp!68zVyJ9dlxVLX0r7KnV8SHjIQVnctJR9SQFvDPooHJwbOI3SXiVFMwQ8bfAFb7DNxl92Qg$) and

<https://journals.plos.org/plosone/s/file?id=ba62/PLOSOne_formatting_sample_title_authors_affiliations.pdf>

*Thank you for this note.*

2. Thank you for including your ethics statement: "We obtained institutional review board (IRB) approval from the University of California San Francisco Committee on Human Research, the University of California Berkeley Committee on Protection of Human Subjects, the Olive View-UCLA Medical Center IRB, and the Highland Hospital—Alameda Health System IRB to conduct this study with written or verbal consent. ".

Please provide additional details regarding participant consent. In the ethics statement in the Methods and online submission information, please ensure that you have specified what type you obtained (for instance, written or verbal, and if verbal, how it was documented and witnessed). If your study included minors, state whether you obtained consent from parents or guardians. If the need for consent was waived by the ethics committee, please include this information.

Once you have amended this/these statement(s) in the Methods section of the manuscript, please add the same text to the “Ethics Statement” field of the submission form (via “Edit Submission”).

For additional information about PLOS ONE ethical requirements for human subjects research, please refer to [http://journals.plos.org/plosone/s/submission-guidelines#loc-human-subjects-research](https://urldefense.com/v3/__http://journals.plos.org/plosone/s/submission-guidelines*loc-human-subjects-research__;Iw!!LQC6Cpwp!68zVyJ9dlxVLX0r7KnV8SHjIQVnctJR9SQFvDPooHJwbOI3SXiVFMwQ8bfAFb7ADLs7xIQ$).

*Thank you for this note. We have edited our statement and have uploaded the updated version.*

3. We note that you have stated that you will provide repository information for your data at acceptance. Should your manuscript be accepted for publication, we will hold it until you provide the relevant accession numbers or DOIs necessary to access your data. If you wish to make changes to your Data Availability statement, please describe these changes in your cover letter and we will update your Data Availability statement to reflect the information you provide.

*Thank you for this note.*
