# Supplementary material for: Provider and administrator-level perspectives on strategies to reduce fear and improve patient trust in the emergency department in times of heightened immigration enforcement
Source: PLoS One. 2021 Sep 10;16(9):e0256073. doi: 10.1371/journal.pone.0256073 (PMC8432754; doi:10.1371/journal.pone.0256073)
Supplement: Attachment — Submitted filename: Response to ReviewersPONE-D-20-28725.doc [file pone.0256073.s005.doc]

**Additional Editor Comments (if provided):**

The reviewers asked you to revise the manuscript and to address some other comments. In addition, you need to revise the paper by using English editing service or professional expert to ensure consistency and clarity of the manuscript before further consideration.

*Thank you for this comment. An editor has reviewed the manuscript in detail.*

**Reviewer 1:**

The authors have satisfactorily addressed all of my comments. Just two notes:

1. I disagree with many of the comments from Reviewer 2, and I believe some of the edits the authors made to address R2's concerns did not improve the paper.

2. In the limitations section, the authors comment on the generalizability of their findings, but it may be worth noting that qualitative work rarely aims at generalizability but rather at transferability.

*Thank you for this comment. We have changed the language in the limitations section to reflect transferability, rather than generalizability; and have responded to comments from all reviewers, including reviewer 2.*

**Reviewer 5:**

Nice job on an important subject. Well written with unique methodology. I had minimal comments which have been addressed. The manuscript has been made even more relevant and generalizable internationally.

*Thank you for this comment.*

**Reviewer 6:**

Carolina Ornelas and colleagues reported a study on "Policies and protocols on reducing undocumented patients’ fear in the emergency department”. They recruited and interviewed 41 ED providers and administrators from three California Eds, and found out existing policies and recent policy changes that facilitate access to care for undocumented patients; limited training and communication around policies; significant uncertainty across provider-types on policies, laws, and the roles of staff; providers stated they are taking an active role in building safety and trust and see their role as supporting undocumented patients. The authors conclude that there is a need for active, multi-disciplinary ED policy training, clear policy details including the extent of providers’ roles, protocols on the screening and documentation of status, and continual reassessment of our health systems to reduce fear and build safety and trust with our undocumented communities. Overall, the manuscript is professionally written and the study is designed well. However, I have one concern in policy and protocol recommendations that I will try to explain below.

Nearly a quarter of the nation’s undocumented immigrants reside in California, most of them are from Latin America, most of the others are from Asia. Multiple studies suggested that Latino immigrants facing the most discrimination in the USA, and COVID-19 fueling anti-Asian racism. Undocumented immigrants are even more vulnerable to experiences of discrimination under COVID-19 pandemic. Racism and Discrimination has been identified as a major stressor and influence on immigrant health. Undocumented immigrants are fearful of seeking medical attention because of not only the risk of exposing them to immigration enforcement but also being stigmatized and discriminated against by nonimmigrant-friendly medical providers. I think anti-racism and discrimination education of the training is needed in protocol recommendation, and could you please add this aspect in the discussion.

*Thank you for this recommendation. We have included brief commentary on anti-racism training in the background, discussion, and policy recommendations.*
